# Impairment of vascularization of the surface covering epithelium induces ischemia and promotes malignization: a new hypothesis of a possible mechanism of cancer pathogenesis

**DOI:** 10.1007/s12094-014-1255-x

**Published:** 2014-11-19

**Authors:** A. I. Karseladze

**Affiliations:** Department of Pathology, N. N. Blokhin Russian Cancer Research Centre, Academy of Medical Sciences of the Russian Federation, Kashirskoe sh. 24, 115478 Moscow, Russia

**Keywords:** Angiogenesis, Tumor angiogenesis, Ischemia in carcinogenesis, Epithelial–stromal interface, Cancer pathogenesis

## Abstract

**Purpose:**

To study the peculiarities of vascularization at the stromal–epithelial interface in different types of epithelia and their alterations in precancerous lesions.

**Materials and methods:**

Peritumoral tissues of 310 patients, tissues of 180 healthy persons and of 50 human embryos and fetuses were used. Traditional histological as well as immunohistochemical methods have been used.

**Results:**

The study reveals that the occurrence of blood capillaries in surface squamous epithelium is an ordinary event, both in healthy persons and in peritumoral regions of the patients with squamous cell carcinoma. Glandular epithelial coverings, as well as transitional epithelium, do not contain blood vessels. In squamous epithelium, only basal cells are in contact with the membrane and underlying stroma, the cells of the upper layer receiving nutrients through diffusion. Thus, the cells of squamous epithelium are more vulnerable to blood deficiency, since for instance in the pseudo-multilayered respiratory epithelium each cell is attached directly to the basal membrane and has more ample access to the blood supply. Metaplastic squamous epithelium has a markedly reduced vascularization and seems to be more sensitive to carcinogenic stimuli. High-grade dysplastic squamous epithelium and carcinoma in situ do not contain blood vessels.

**Conclusion:**

The process of redistribution of vascular network occurring at the interface of epithelial–stromal frontier plays an important role in maintaining the adequate metabolism of cells including those of epithelial covering. Impairment of this mechanism most probably promotes precancerous alterations.

## Introduction

A vast amount of literature is devoted to the investigation of the early stages of malignization, especially of the covering epithelium. The literature deals with the problems of viral and chemical carcinogenesis, and in recent decades the genetic and molecular disturbances in epithelial cells. We do not wish to diminish the importance of the above-mentioned factors in the etiology of cancer but, as pathologists, we have to acknowledge the existence of serious gaps in our understanding of the pathogenesis of malignant growth. In fact, one of the most reasonable questions that arises in the process of assessing different factors responsible for malignization of the cell is: how can we explain the existence of different rates of mutation penetrance, the capacity to eliminate oncogenic viruses from cells and other facts which favor the resistance of cells to carcinogenic stimuli? The latter is probably the result of coaction of the adaptational mechanisms, both of intracellular structural and functional type, and external environmental ones.

One of the most important environmental factors is vascular microcirculation under normal circumstances as a part of the protective mechanisms in the process of malignization, which unlike the phenomenon of angiogenesis in the stroma of tumors [1, 2] has not yet been investigated.

The process of induction of angiogenesis in the stroma of tumors starts at relatively late stages of oncogenesis. Information about the possible events which occur in the vascular network at earlier, precancerous stages is practically absent.

Nevertheless, several preliminary communications have been published in which the authors have persuasively demonstrated the occurrence of blood vessels in the covering of various types of epithelium in normal and different pathological entities, mainly of a non-neoplastic nature, although predominantly in animals.

In many species of the animal world, the occurrence of blood vessels in the epithelium is quite an ordinary phenomenon. For instance, the epidermis of amphibians contains many capillaries, especially in the foci rich with keratinocytes [3]. Many blood vessels are surrounded by connective tissue in the pseudo-multilayered ciliary epithelium of the oral mucosa of the cane toad [4]. The vomeronasal neuroepithelium is also vascularized in rats [5], and intraepithelial blood vessels can be detected in the nasal mucosa of dogs [6]. The fact that blood vessels occur in the retina is well known [7]. The same authors [8] supposed that the penetration of blood vessels into rat retina induces polarization of the cells adjacent to the capillaries.

It is of interest to note that the appearance of capillaries in the surface epithelium of several organs is found to be a routine event at specific stages of embryogenesis. For example, Sangari et al. [9] observed intraepithelial capillaries in the neuroepithelium of human embryos at 12–24 weeks of gestation, although they gradually disappear in later stages.

As far as human pathology is concerned, the phenomenon of blood vessel penetration into the covering epithelium is well documented in the esophageal wall of patients with portal hypertension [10], in vulvar dermatosis [11], and in pharyngeal tonsils [12]. In the pterygial epithelium, Seifert and Sekundo [13] found capillaries with perivascular connective tissue in 11 out of 26 patients, and interpreted the findings as a reaction to hypoxia or deficiency of any other substances transported via the bloodstream. The authors hypothesized that the fibroblasts may contribute to the pathological dedifferentiation of the conjunctival epithelium.

The interpretation of the nature of the vascular network in epithelium is not always easy. For example, Grosshans et al. [14] encountered capillaries without surrounding pericytes in the epithelium of the glans penis. They imagined that this might be intraepithelial capillary hemangioma.

There are little data on the presence of lymphatic vessels at preinvasive stages of oncogenesis [15–17]. Some authors have documented the growth of lymph vessels in the conjunctiva at the preinvasive stage of melanoma [18].

## Materials and methods

The study involved the investigation of histologic slides of 310 patients with tongue, cervical, laryngeal (all locations), esophageal (all cases: middle thoracic part), lung, penile, vulvar and vaginal squamous cell carcinoma and transitional cell carcinoma of the bladder (Table 1). All patients received some type of adjuvant therapy; the majority chemo- and radiotherapy with a mean interval of 2–3 months before surgery. Eleven patients with penile carcinoma had phimosis: one of whom had undergone surgical removal 20 years earlier at the age of 21. Some penile carcinomas were recurrent neoplasms after laser-, chemo- or radiotherapy. Many cervical carcinomas were diagnosed after repeated biopsies.

**Table 1:** Characteristics of the clinical material

No.	Site	Number of cases	Gender		Age (range)	Age (modal)
			M	F		
1	Tongue	35	22	13	25–81	50–69
2	Larynx	21	17	4	41–80	50–59
3	Esophagus	33	27	6	29–75	50–70
4	Penis	26			39–90	50–70
5	Cervix	49			27–71	30–49
6	Vagina	3			40, 52, 64	
7	Vulva	46			34–82	60–80
8	Lung	53	45	8	41–90	50–69
9	Bladder	44	38	6	41–88	50–69

In addition to the above-mentioned material, we also investigated the tissues of 50 embryos and fetuses of 8–40 weeks of gestation.

All tumors except bladder were squamous cell carcinoma with different degrees of malignancy. Tumors of the bladder comprised transitional cell carcinomas of various gross characteristics. Three patients had foci of epidermization, 9 had poorly differentiated transitional cell carcinoma, and 10 had non-invasive carcinoma.

As a control, we investigated 180 cases of corresponding healthy organs (20 cases each) removed during surgical interventions for non-neoplastic pathology, traumas.

Slides, 5 µm thick, of formalin-fixed, paraffin-embedded material were used for the study. Taking into consideration the possibility of artificial results obtained on the tangentially cut slides, very often we performed additional serial cuts. In some cases, we even dismounted the specimen, re-embedded it perpendicular to the initial position axis and prepared new set of slides.

In addition to traditional staining methods (H&E, Van-Gieson), immunohistochemical stainings with CD31 (clone BC2, Biocare, dilution 1:700), CD34 (clone QBND/10, Dako, diluted 1:700) Factor VIII (clone Rb, BioCare dilution 1:700), Podoplanin (D2-40, Dako, dilution 1:350) were performed for identification of the blood and lymphatic vessels.

## Results

**Fig. 1:**
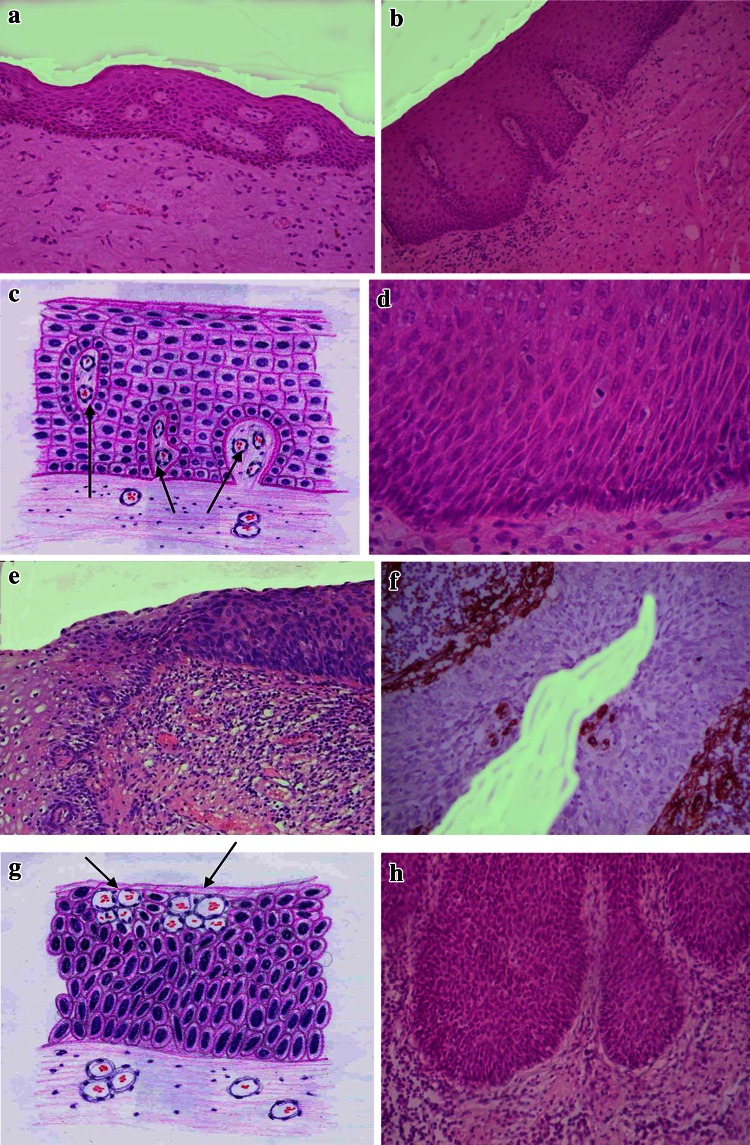
All slides except immunohistochemical reactions are stained with Hematoxylin and Eosin. **a** Blood capillaries surrounded by stroma in the normal squamous epithelium of the patient with vulvar carcinoma. Original magnification ×100. **b** Successive stages of sequestration of the stroma with blood capillaries in patient with carcinoma of the tongue. Original magnification ×120. **c** Schematic representation of the sequestration of the stroma with blood capillaries. *Arrows* indicate blood vessels with surrounding stroma. **d** Absence of capillaries in the focus of high-grade dysplasia in patient with esophageal carcinoma. Original magnification ×200. **e** Contrast between vascularized normal squamous epithelium (*on the left*) and dysplastic epithelium devoid of blood vessels (*on the right*) in patient with cervical carcinoma. Original magnification ×160. **f** Capillaries in the superficial layers of the epithelial covering over dysplastic epithelium. Patient with cervical carcinoma. Immunostaining for CD34. Original magnification ×160. **g** Schematic representation of the previous (1 g) figure. *Arrows* indicate the blood vessels. **h** Absence of capillaries in the focus of vulvar carcinoma in situ. ×160

The study of the distribution of blood vessels in patients with squamous carcinoma as well as in healthy persons revealed that the normal peritumoral squamous epithelial covering is always vascularized. Capillaries are encountered in the epithelium regardless of its thickness, and may be seen even in very thin linings (Fig. 1a).

Throughout the epithelium, the vessels are randomly distributed at all levels, having both vertical and horizontal orientations.

Reconstruction of the vascularization processes of the squamous epithelial covering on consecutive and serial cuts shows that in fact the process is the changing of the relief of subjacent stroma. The latter penetrates in the form of excrescences and sprouts into the covering epithelium capturing the blood vessels (Fig. 1b, c). On the contrary, there is sometimes an impression that the proliferating basal cells of the epithelium invaginate deeper, enfold the underlying stroma and incorporate blood capillaries. So what we perceive on the histological slides as intraepithelial blood vessels is a reflection of alterations on the epithelial–stromal interface.

All the above-mentioned describes the angiogenic patterns in the covering epithelium without visible signs of precancerous alterations. We found that as soon as dysplastic alterations start, the vascular network undergoes important changes.

Comparison of normal and dysplastic epithelial vascularization showed that the fully developed, high-grade dysplastic foci do not contain capillaries (Fig. 1d). As a result of this lack of blood vessels, dysplastic foci contrast sharply with the neighboring normal epithelium (Fig. 1e).

The disappearance of blood vessels is a gradual process; their amount decreases as the dysplasia progresses from low to high grades. Foci represented by a slight degree of dysplasia still contain capillaries predominantly in the basal layers of the epithelium or even in the upper externally normal layers (Fig. 1f, g).

No blood vessels were observed in the foci of in situ carcinoma (Fig. 1h). In those rare cases where the carcinoma in situ structures were vascularized, either they were indistinguishable from incipient invasive carcinoma, or the capillaries were localized to the uppermost layers of the epithelium and thus may be of a residual nature.

All the above findings correspond to the peculiarities of the vascularization of the squamous epithelium. Unexpected results were obtained from the analysis of the glandular (respiratory) and transitional types of epithelium.

**Fig. 2:**
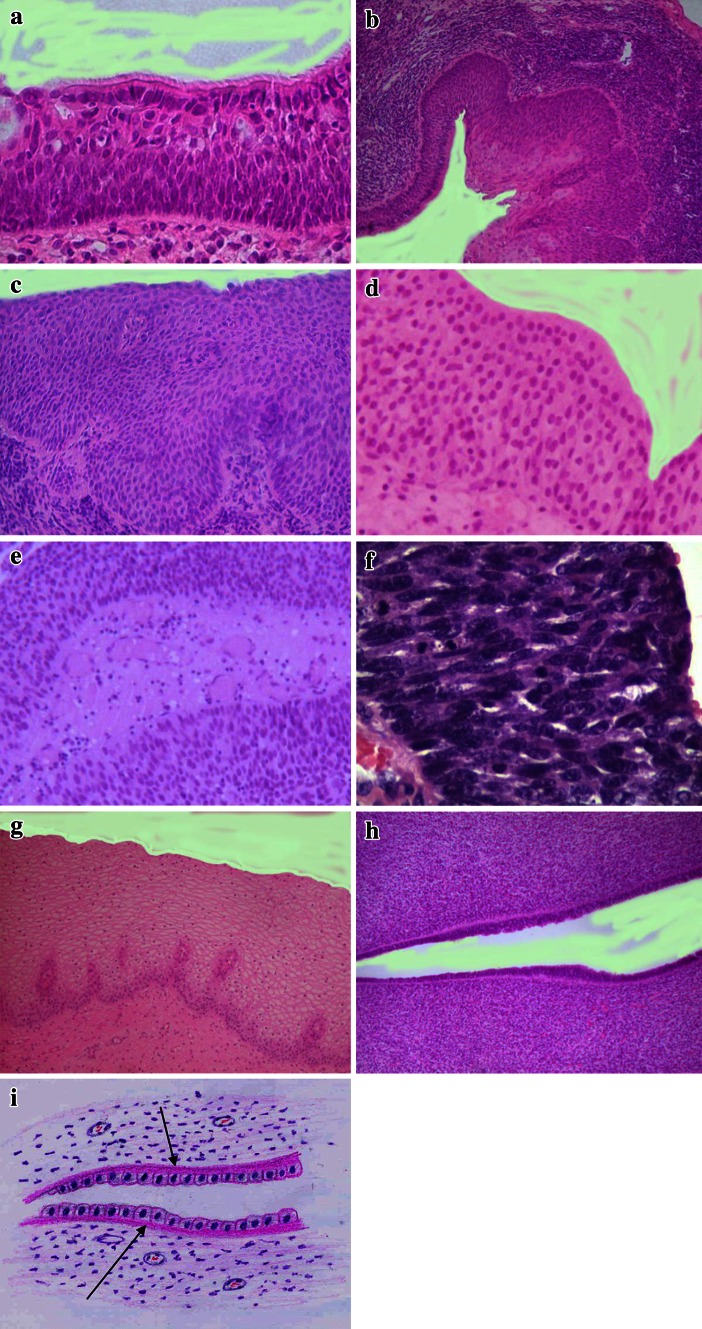
All slides except immunohistochemical reactions are stained with Hematoxylin and Eosin. **a** Normal respiratory epithelium in the bronchial lining without blood vessels. The cells are separated from the stroma by eosinophilic membranous substance. Patient with lung cancer. Original magnification ×160. **b** Transition from normal respiratory epithelium to the metaplastic squamous epithelium in the bronchial lining. Original magnification ×120. **c** Penetration of capillaries in the metaplastic squamous epithelium in the bronchial wall of the same patient Original magnification ×200. **d** Absence of blood capillaries in the normal transitional cell lining of the bladder. Original magnification ×160. **e** Stalk of the bladder papilloma rich in capillaries. Original magnification ×120. **f** Carcinoma in situ of the bladder without blood vessels. Original magnification ×200. **g** Blood capillaries in the vagina of the fetus of 24 weeks of gestation. Original magnification ×120. **h** Eosinophilic membranous substance under the glandular lining of the future uterus in embryo of 23 weeks of gestation Original magnification ×100. **i** Schematic drawing of the same figure (Fig. 2h). The layer of the membranous substance is indicated by *arrows*

No capillaries were found in the normal respiratory lining epithelium of any cases of squamous cell lung carcinoma regardless of the thickness of the epithelium (Fig. 2a).

Of special interest is the secondary metaplastic epidermization in the glandular epithelium. Metaplastic foci showed different rates of vascularization, low in the lung, and in the larynx (Fig. 2b, c) but in the penile glands in the surroundings of the cancer the foci of epidermization always had a rich vessel network and were clearly visible within the lining of the same gland.

The pattern seen in transitional epithelium of the bladder and transitional cell carcinomas differed from that observed in the squamous and respiratory epithelium.

Normal transitional epithelium does not contain blood vessels (Fig. 2d). Very seldom a rich capillary network may be encountered in the basal layers, mainly in patients with some circulatory disturbances or inflammations.

Blood vessels do penetrate the transitional epithelial lining of the bladder as carcinogenesis starts, but as a rule the process runs parallel to the growth of exophytic tumors. Blood vessels are one of the components of the mesenchymal stalks containing blood capillaries, perivascular stroma with stromal cells, etc. which make up the architectural body for the future exophytic papillary tumors (Fig. 2e).

As far as flat lesions of the bladder are concerned, foci of dysplasia, carcinoma in situ do not contain any capillaries at all (Fig. 2f).

The epithelial coverings of healthy persons in the control group showed the same characteristics as those of cancer patients: squamous epithelium is vascularized, glandular covering is avascular. No parallelism between the amount of blood vessels in the stroma and the corresponding epithelial covering, or emergence of blood capillaries in the metaplastic epidermoid foci was found.

To discover any peculiarities of the epithelium vascularization at the frontier of the stroma, we paid attention to the fact that the respiratory epithelium, unlike squamous, is separated from the underlying tissues by broad amorphous eosinophilic material (Fig. 2a). This material also disappears in the foci of epidermization of the respiratory epithelium in non-cancer patients.

In embryos and fetuses, squamous epithelium almost always contains blood vessels, at least in the basal layer of the covering. The existence of blood capillaries may be detected very early at 12 weeks of gestation (in the vaginal wall, skin etc.) (Fig. 2g). Glandular epithelium does not contain blood vessels in the gestational period.

As in the postnatal period, the embryonal glandular epithelium is linked to the underlining stroma by a thick eosinophilic material under the basal membrane (Fig. 2h, i). This is clearly visible in the linings of the female genital tract, with a sharp divide between future utero-cervical, nonvascularized epithelium on underlying thick eosinophilic membranous structures, and the squamous future vaginal epithelium rich in blood capillaries without amorphous material.

## Discussion

Our results seem to a certain extent obvious, but their association with the processes of carcinogenesis did not attract much attention probably due to the assumption that some of these structures are rather artifacts, although nobody has tried to from whence comes the dynamic changes of blood vessel movement and regular character of these patterns? Coexistence of normal epithelial squamous covering containing blood capillaries and adjacent high-grade dysplasia without blood vessels in the same field of vision cannot be the result of tangential cuts or artifacts.

Data on the antenatal development in human embryos and fetuses show that the penetration of blood vessels (of stromal excrescenses) into the epithelial covering often occurs in several organs, but towards the end of gestation this capacity is restricted, though it may recapitulate in the postnatal period. The appearance of blood capillaries in the developing antenatal organs cannot be ascribed to methodological errors either.

It is worth mentioning that the reduction of the epithelial vascularization at the end of the antenatal period coincides with the emergence of thick eosinophilic amorphous material under glandular epithelial coverings.

**Fig. 3:**
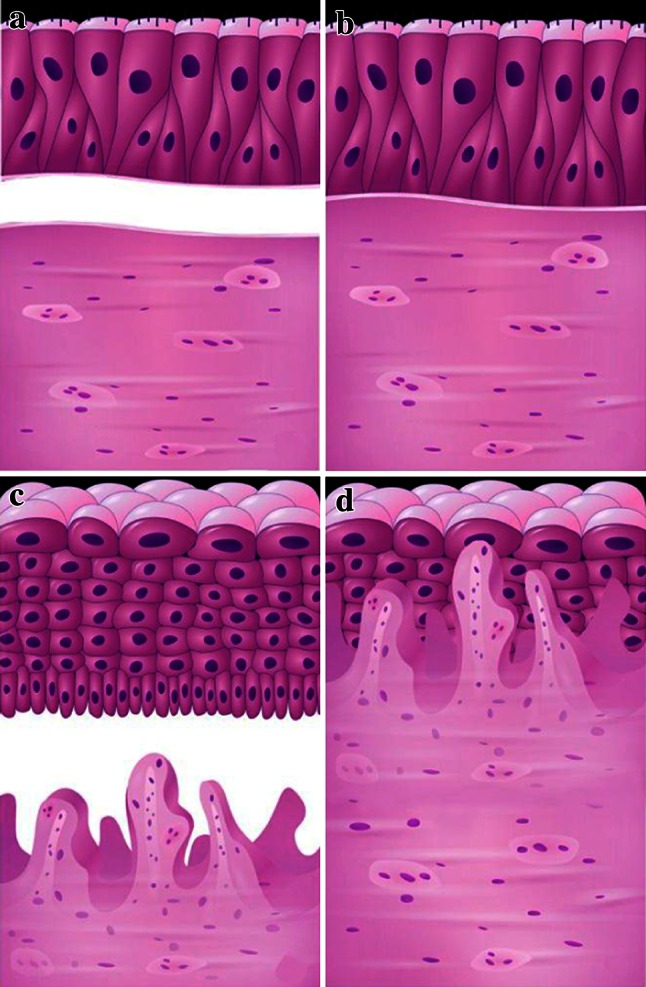
Schematic representation of the relationship between different types of epithelium and underlying stroma. **a**, **b** In the pseudo-stratified respiratory covering, all cells are attached to the basal membrane and receive equal blood supply. There is no need for additional ingrowth of blood vessels into epithelium. **c**, **d** Squamous epithelium is a true stratified structure; only basal cells are attached to the membrane and thus have access to the stromal blood capillaries. The rest of the cells compensate for the lack of sufficient nutrients through the additional close contacts with the intraepithelial blood vessels which accompany the stromal excrescences

**Fig. 4:**
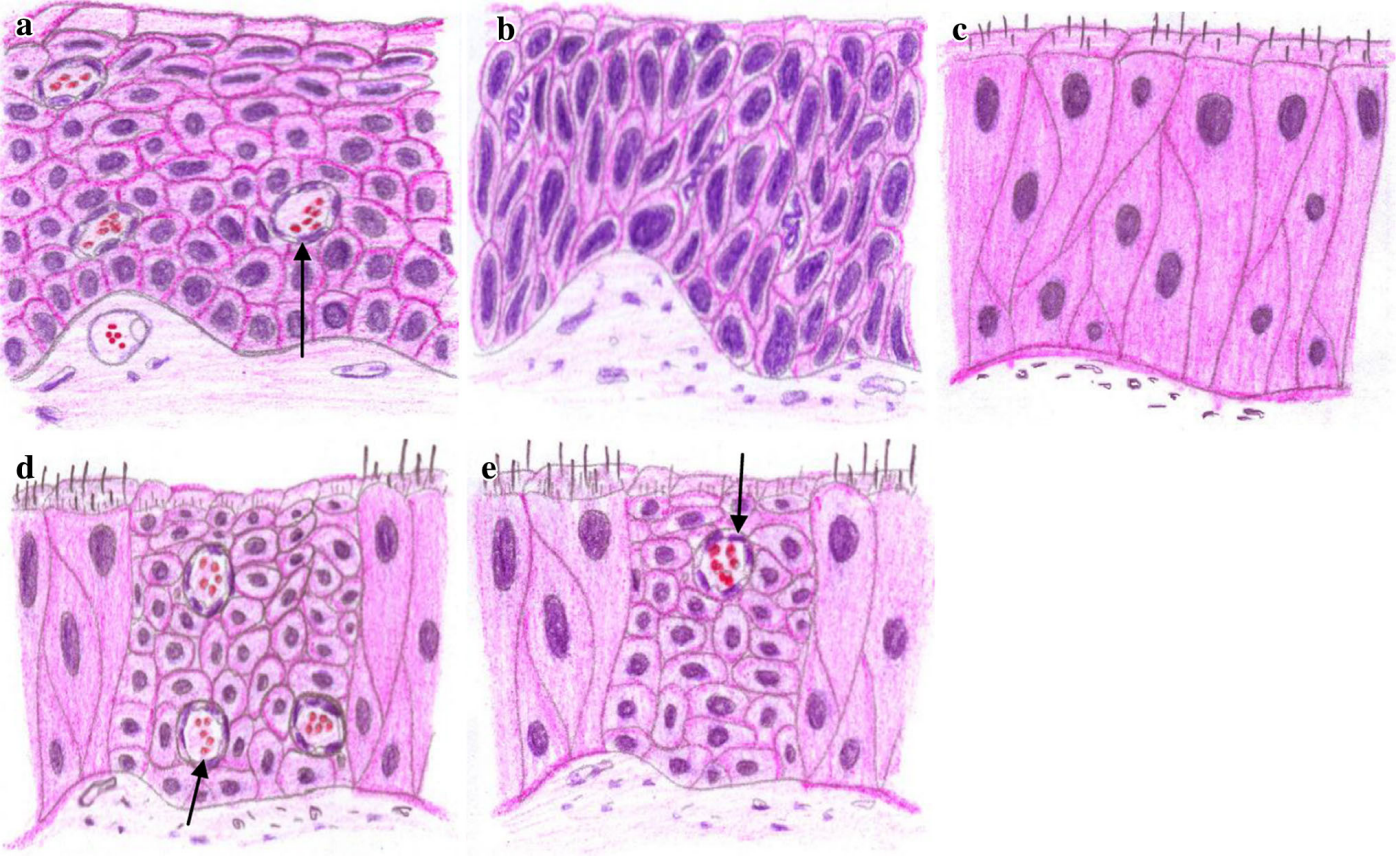
Normal squamous epithelium **a** is vascularized but the foci of fully developed dysplasia **b** do not contain blood capillaries at all. The foci of squamous metaplasia in the respiratory epithelium **c** need transformation of the blood supply network. The amount of blood capillaries invading metaplastic epithelium **d**, **e** may vary from case to case and result in different levels of blood flow (*arrows* blood vessels)

It is well known that the respiratory epithelium is pseudo-multilayered, each cell attached to the basal membrane and in direct contact with stromal blood circulation (Fig. 3a, b). On the contrary, squamous epithelium is a true multilayered structure, where only basal cells are in contact with the membrane and the cells of the higher layers receive blood nutrients through diffusion; hence the necessity of developing excrescences, which facilitate the delivering of blood vessels to the superficially located cells of the epithelial covering (Fig. 3c, d). Reduction of the amount of blood vessels in the squamous epithelium most probably impairs normal metabolism and promotes dysplastic alterations. This may explain one of the most interesting findings—the absence of blood vessels in the high-grade dysplastic epithelial layers (Fig. 4a, b). In typical cases, this is well demonstrated at the border of normal and dysplastic epithelium. There are two possible explanations: dysplastic epithelium may suppress angiogenesis (stromal proliferation), or primarily the blood supply is reduced, lowering the proper level of nutrition thereby inducing or promoting dysplasia. The latter assumption seems more justified since it is favored by a certain parallelism in the gradual increase of dysplastic alterations and decrease in the rate of vessel density. Besides, several well-documented investigations show that hypoxia is one of the most important environmental factors in the process of carcinogenesis [19]. It favors activation of c-Myc, VEGF receptors and production of biologically active proteins (HIF-1α, Glut-1 and CA9), which promote malignization [20–22].

Metaplasia and epidermization of the glandular epithelium change the pattern of blood supply in the metaplastic epithelium towards the reduction of normal metabolism since metaplastic foci generally have a sparse vascular network (Fig. 4c–e) and seem more vulnerable to carcinogenic stimuli.

Vascularization pattern influences morphogenetic processes either. Transitional surface epithelium does not contain blood vessels, but as mentioned earlier, the penetration of blood vessels into this type of epithelium runs parallel with the building up of the papillary neoplasms (both benign and malignant) and is accompanied by a pronounced connective tissue component. This type of vascular architecture with marked perivascular stromal cuffs and high capillary density probably guarantees sufficient blood supply to maintain a normal metabolism in the cells of papillary tumors, whereas the metabolism is definitely dysregulated in flat lesions—high-grade dysplasia and carcinoma in situ devoid of capillary network.

These alternative vascularization patterns of early stage malignancies in the bladder mucosa may determine the morphogenesis of future urothelial tumors.

We believe that the specific characteristics of the basal membrane may play an important role in the regulation of the capillary network. This assumption is supported by recent data from several authors who have demonstrated that the chemical composition of the basal membrane, of its various components especially proteins, favors the differentiation of the endothelium, converting the individual endothelial cells into tube-like structures [23]. It seems logical that the different chemical composition of the basal membrane induces different morphogenetic events at the stromal–epithelial junction.

## Conclusion and future perspectives

Under normal physiological conditions, each type of epithelium has its own specific form of contact between the cells and subjacent stroma to maintain maximal access to nutrients for all the cellular population.

In squamous epithelium, only basal cells are in direct contact with the stroma, the rest of the cells receive blood by the penetration of stromal excrescences upward into the covering.

In respiratory epithelial coverings, each cell is in contact with the stroma, and thus has direct access to the blood supply. There is no need for stromal ingrowths into the epithelium.

In dysplastic alterations, the boundary between squamous epithelium and stroma becomes flat, the number of stromal ingrowths (resp. blood vessels) is markedly reduced, and as a consequence the epithelial cells suffer ischemia.

Similarly, the foci of squamous metaplasia in respiratory epithelium lack an adequate capillary network and serve as a substrate of malignization in the initially well-vascularized glandular epithelium.

Looking to the future, we must investigate the reasons for the remodeling of the stromal–epithelial relationship in the process of malignization- (increasing dysplasia of the covering epithelium) and devise therapeutic approaches for the restoration of an adequate vascularization or nutrient supply.

In the future, the above-mentioned data may be used in diagnostic processes as well The dynamics of the gradual disappearance of intraepithelial blood capillaries could serve as an indicator of the precise rate of dysplasia and may also serve as an objective differential diagnostic tool in excluding reactive polymorphism in epithelial coverings.
